# Creative arts therapies for stroke patients: A systematic review and meta-analysis protocol

**DOI:** 10.1371/journal.pone.0341629

**Published:** 2026-01-23

**Authors:** Vikram Arora, Alex Thabane, Jude Hynes, Adam Sutoski, Mohit Bhandari

**Affiliations:** 1 Department of Health Research Methods, Evidence, and Impact, McMaster University, Hamilton, Ontario, Canada; 2 Temerty Faculty of Medicine, University of Toronto, Toronto, Ontario, Canada; 3 Department of Psychology, University of Western Ontario, London, Ontario, Canada; 4 Faculty of Medicine and Health Sciences, McGill University, Montreal, Québec, Canada; 5 Department of Surgery, McMaster University Medical Center, Hamilton, Ontario, Canada; University of Toronto, CANADA

## Abstract

**Introduction:**

Stroke is a leading cause of long-term disability and mortality worldwide. Survivors can experience a range of physical and emotional challenges, often leading to depression, anxiety, and a poorer quality of life. Creative arts therapies (CATs), an umbrella term encompassing music, art, dance/movement, drama, and creative writing therapies, have increasingly been explored in stroke survivor populations as interventions to improve psychological outcomes. Qualitative analysis suggests these therapies can be helpful, but the exact efficacy of CATs in stroke rehabilitation, as well as the optimal intervention types and treatment protocols, has yet to be established. This systematic review and meta-analysis plans to evaluate the effect of CATs on depression, anxiety, and quality of life among adults recovering from stroke.

**Methods:**

This protocol has been prospectively registered with PROSPERO (CRD420251237926). Eligible studies will include primary quantitative research involving creative arts interventions. Searches will be conducted in Medline, Embase, and PsycInfo from inception to December 2025. Two reviewers will independently screen records, extract data, and assess study quality and the certainty of the evidence using the RoB 2, ROBINS-I, and GRADE tools. Restricted maximum likelihood random-effects meta-analyses of Cohen’s *d* effect sizes and risk ratios will be performed to calculate pooled effect sizes for each outcome. Subgroup analyses will explore moderators such as the effect of study design, intervention type, session frequency, and patient setting.

**Dissemination of results:**

Results will be disseminated through a peer-reviewed publication, conference presentations, and clinical networks to inform evidence-based guidelines on the use of CATs in multidisciplinary stroke care.

## Introduction

Stroke is a leading cause of death and long-term disability worldwide [1]. Survivors often face a range of physical impairments, including motor and speech limitations; roughly 40% of survivors have moderate-to-severe physical disability in the years following their stroke [2]. In addition to these physical challenges, stroke survivors frequently experience psychological distress, with approximately one-third developing post-stroke depression [3–5] and one-quarter developing anxiety disorders [6]. These mood disturbances are more than just emotional challenges; they are associated with poorer functional recovery, reduced social participation, and diminished quality of life [7]. Despite this, depression and anxiety after stroke continue to be underrecognized and undertreated [8,9].

Given the negative impacts of stroke on mental well-being and quality of life, there has been a growing interest in holistic interventions to support stroke patients’ recovery beyond standard medical and physical rehabilitation [10]. One promising avenue is the use of creative arts therapies (CATs), which are therapeutic interventions such as music, visual arts, dance/movement, drama, or creative writing, to achieve health benefits [11]. In stroke rehabilitation, these creative modalities have been explored as complementary treatments to address the psychosocial needs of survivors. Notably, a randomized controlled trial demonstrated that stroke patients who participated in a multimodal creative arts program alongside conventional physiotherapy showed significantly lower depression levels, better quality of life, and improved physical function compared to those receiving physiotherapy alone [12]. Such findings highlight the potential for CATs to enhance both mental and physical aspects of recovery, motivating further investigation into their efficacy for post-stroke emotional health.

There have been several literature reviews evaluating the literature on the use of CATs for stroke survivors. A recent scoping review of creative activities in stroke rehabilitation identified various art-based interventions used in this population, and found evidence of positive impacts on patients’ daily living activities, limb motor function, fine motor skills, and emotional well-being [10]. Additionally, a qualitative systematic review synthesized stroke survivors’ experiences with creative arts-based therapies, reporting numerous benefits across physical, psychological, social, and even spiritual domains [13]. These reviews collectively suggest that CATs are a promising adjunct in stroke care. However, the extent to which CATs benefit stroke patients remains largely unknown, as no meta-analysis of the available literature has yet been conducted. Conducting a meta-analysis will allow us to estimate the strength of the effect of CATs on anxiety, depression, and quality of life using data from the entire scientific literature, rather than relying on individual findings that may be underpowered or inconsistent [14]. It will also enable quantitative exploration of subgroup effects, such as differences by intervention type (e.g., music vs. art vs. dance therapy), session frequency or duration, and treatment setting (inpatient vs. outpatient), which may moderate therapeutic outcomes in related rehabilitation contexts. Coupled with a certainty of the evidence assessment using the Grading of Recommendations Assessment, Development and Evaluation (GRADE) framework, a new, comprehensive meta-analysis will enable the generation of robust conclusions to inform clinical decision-making. Taken together, we have designed a systematic review and meta-analyses exploring the efficacy of CATs for improving anxiety, depression, and quality of life in stroke patients. Such evidence will inform clinicians and rehabilitation specialists about the potential benefits of CATs and guide the potential integration of creative approaches into stroke care.

## Methods

We have prospectively registered this protocol with PROSPERO (CRD420251237926) and reported it in accordance with the Preferred Reporting Items for Systematic Review and Meta-Analysis Protocols (PRISMA-P) guidelines (S1 File) [15].

### Study objectives

The primary objective is to evaluate the efficacy of CATs in improving anxiety, depression, and quality of life in stroke patients. The secondary objective is to explore subgroup effects for the following characteristics: session frequency (e.g., single vs. multi-session), intervention type (e.g., art vs. music vs. other types of CATs), and treatment setting (i.e., inpatient vs. outpatient).

### Outcomes

The primary outcomes of interest will be anxiety, depression, and quality of life. Anxiety is defined as an “uncontrollable, diffuse, unpleasant, and persistent state of negative affect, characterized by apprehensive anticipation regarding unpredictable and unavoidable future danger, and accompanied by physiological symptoms of tension and a constant state of heightened vigilance” [16]. Depression is defined as a mood disorder marked by feelings of sadness, emptiness, or irritability, along with somatic and cognitive changes that substantially impair an individual’s ability to function [17]. Quality of life is defined according to the World Health Organization as an “individual’s perception of their position in life in the context of the culture and value systems in which they live and in relation to their goals, expectations, standards and concerns” [18].

### Selection criteria

Studies must meet the following eligibility criteria to be included: (1) primary research studies; (2) involve human participants who have experienced ischemic or hemorrhagic stroke, as defined by the updated definition for strokes [19]; (3) include an intervention involving creative arts therapy—such as art therapy, music therapy, dance/movement therapy, drama therapy, or poetry/creative writing [11]; (4) report quantitative data for at least one relevant outcome for our study (depression, anxiety, or quality of life); and (5) be published in a peer-reviewed journal. The exclusion criteria will be: (1) conference proceedings, abstracts, protocols, reviews, or theses; and (2) studies not reporting quantitative outcome data. Any study that is not published in English will be translated using Google Translate.

### Search strategies

We plan to systematically search the Medline, Embase, and PsycInfo databases from inception to December 3, 2025 for studies that meet our eligibility criteria. For each database, we have developed tailored search strategies by combining Medical Subject Headings (MeSH) (e.g., hemorrhagic stroke, ischemic stroke) and keywords (e.g., creative arts therapy, music therapy, art therapy) using the Boolean operators AND/OR. The full search strategy for each database is included in S2 File. Additionally, we will conduct a manual search of the reference lists of all included studies and relevant review articles [10,13] to identify any additional eligible studies that may not have been captured in the initial database search.

### Screening

Two independent reviewers will independently and blindly screen the titles and abstracts of all records identified through the search. Any disagreements will be resolved through discussion and if consensus cannot be reached, a third reviewer will be consulted. Full-text articles will be retrieved for studies deemed potentially eligible. The same dual-reviewer process will be applied to the full-text screening stage. Screening will be managed using the Covidence platform (Melbourne, Victoria, Australia) [20]. The results of the study selection process will be presented using a PRISMA flow chart.

### Data extraction

We will develop a standardized data extraction template to extract relevant data from eligible studies. Two reviewers will independently extract the data, with any discrepancies resolved by discussion to achieve consensus or adjudication by a third reviewer. Extracted data will include study characteristics (i.e., author, year of publication, study design, location of study), patient demographics (e.g., age, sex), disease characteristics, intervention characteristics (e.g., type, number of sessions, duration of therapy), and outcome data for the outcomes of interest. Only data explicitly reported in the published articles will be extracted. For studies with missing or unclear data that preclude inclusion, we will attempt to contact the corresponding authors to obtain the necessary information; if the data remains unavailable, the study will be excluded from that specific analysis.

### Study quality & certainty of evidence assessment

The risk of bias for included studies will be assessed using established and validated tools appropriate to the study design. For randomized controlled trials, we will use the Cochrane Risk of Bias 2 (RoB 2) tool [21]. RoB 2 is a domain-based evaluation that assesses potential bias across five key domains: bias arising from the randomization process, deviations from intended interventions, missing outcome data, measurement of the outcome, and selection of the reported result. Each domain will be rated as “low risk,” “some concerns,” or “high risk” of bias, and an overall judgment will be provided for each study outcome. For non-randomized studies of interventions, we will apply the Risk of Bias in Non-randomized Studies of Interventions (ROBINS-I) tool [22]. ROBINS-I assesses bias across seven domains, including confounding, selection of participants, classification of interventions, deviations from intended interventions, missing data, measurement of outcomes, and selection of the reported result. Each domain will be judged as “low,” “moderate,” “serious,” or “critical” risk of bias, or “no information.” Two reviewers will independently assess the risk of bias for all included studies and the certainty of the evidence across studies, with discrepancies resolved through discussion or consultation with a third reviewer. We will summarize the results in tables and figures.

To assess the overall certainty of the evidence across studies, we will apply the Grading of Recommendations Assessment, Development and Evaluation (GRADE) approach [23]. GRADE provides a structured framework for evaluating the quality of evidence for each outcome based on five key domains: risk of bias, inconsistency, indirectness, imprecision, and publication bias. The results of the GRADE assessment will be presented in a GRADE Evidence Profile table.

### Statistical analysis

To meet our primary objective, we will conduct pairwise, restricted maximum likelihood random-effects meta-analyses of Cohen’s *d* effect sizes for each outcome of interest. For any dichotomous scales, risk ratios will be used for each of the outcomes. Study weights for all meta-analyses will be calculated using the inverse variance method. No adjustments (e.g., the Hartung-Knapp Adjustment) will be used. For each individual experiment reporting the outcomes using a continuous scale, we will use the reported Cohen’s *d* effect size; when a Cohen’s *d* effect size is not reported, we will calculate it using the reported means, standard deviations, and sample sizes, or through transformation of reported *t*-test or *F*-test statistics using the function *esc* in R software [24]. We will also assess the statistical heterogeneity within each meta-analysis using the I^2^ statistic, considering an I^2^ greater than 40% to represent substantial heterogeneity [25]. Finally, if more than 10 studies are included in a meta-analysis, we will assess the risk of publication bias via visual inspection of a funnel plot in addition to an Egger’s regression test for any continuous outcomes and a Harbord’s test for any dichotomous outcomes. If significant publication bias is detected (p < 0.05), we will conduct a trim-and-fill sensitivity analysis.

To meet our secondary objective, we plan to conduct four subgroup meta-analyses for each outcome based on the following baseline characteristics: study design (i.e., between-subjects; within-subjects), intervention type (e.g., art therapy; music therapy; other), session frequency (i.e., single session; multi-session), and setting (i.e., inpatient; outpatient). These analyses will only be conducted if each categorical subgroup has at least two studies. Lastly, to assess the robustness of the primary analyses, we plan to conduct a sensitivity analysis for each outcome, including only randomized studies.

We will perform all analyses using R Software (version 4.3.2) via RStudio using the *metagen* and *forest* packages. The pooled Cohen’s *d* effect size and risk ratios from all meta-analyses, with the associated 95% confidence intervals (95% CI) will be reported narratively and in forest plots.

### Study timeline

This review is scheduled to begin in December 2025, with database searches to be conducted at that time. We plan to complete the two screening phases (i.e., title and abstract, followed by full-text screening) by March 2026. Then, data extraction and the meta-analysis will be complete by the end of May 2026. We plan to prepare the first draft of the manuscript by June 2026, with final submission to a peer-reviewed journal targeted for August 2026.

## Ethics and dissemination

As this study involves only secondary analysis of aggregate data from previously published studies, ethics approval is not required.

We plan to submit the findings for publication in a peer-reviewed psychology or stroke-related journal and present our results at local, national, and international academic conferences. Beyond traditional dissemination, summaries may also be shared within clinical networks and rehabilitation centres to inform evidence-based practice. Ultimately, this review seeks to strengthen the scientific foundation for integrating CATs into multidisciplinary stroke care, enhancing psychological well-being and recovery outcomes for survivors.

## Conclusion

This systematic review and meta-analysis aims to quantitatively synthesize the scientific literature examining the effects of CATs on anxiety, depression, and quality of life among stroke survivors. In doing so, this study will help determine the therapeutic value of CATs in stroke rehabilitation, which will subsequently inform clinical decision-making.

## Supporting information

S1 File PRISMA-P 2015 checklist. (PDF)

S2 File Search strategy for databases. (PDF)
