# Supplementary material for: Creative arts therapies for stroke patients: A systematic review and meta-analysis protocol
Source: PLoS One. 2026 Jan 23;21(1):e0341629. doi: 10.1371/journal.pone.0341629 (PMC12829804; doi:10.1371/journal.pone.0341629)
Supplement: S2 File — (PDF) [file pone.0341629.s002.pdf]

S2 File: Search Strategy for Databases

Ovid MEDLINE(R) ALL

1 exp Stroke/ or exp Ischemic Stroke/ or exp Hemorrhagic Stroke/ or exp Stroke Rehabilitation/

2 (stroke or ischemi* or hemorrhagi*).ti,ab.

3 Art Therapy/ or Dance Therapy/ or Music Therapy/

4 (visual art or art therapy or creative arts therap* or dance or drama or music therapy or movement therapy or dance therapy or drama therapy or creative arts or creative express*).ti,ab.

5 1 or 2

6 3 or 4

7 5 and 6

APA PsycInfo

1 exp Stroke/ or exp Ischemic Stroke/ or exp Hemorrhagic Stroke/ or exp Stroke Rehabilitation/

2 (stroke or ischemi* or hemorrhagi*).ti,ab.

3 Art Therapy/ or Dance Therapy/ or Music Therapy/

4 (visual art or art therapy or creative arts therap* or dance or drama or music therapy or movement therapy or dance therapy or drama therapy or creative arts or creative express*).ti,ab.

5 1 or 2

6 3 or 4

7 5 and 6

Embase Classic+Embase

1 exp Stroke/ or exp Ischemic Stroke/ or exp Hemorrhagic Stroke/ or exp Stroke Rehabilitation/

2 (stroke or ischemi* or hemorrhagi*).ti,ab.

3 Art Therapy/ or Dance Therapy/ or Music Therapy/

4 (visual art or art therapy or creative arts therap* or dance or drama or music therapy or movement therapy or dance therapy or drama therapy or creative arts or creative express*).ti,ab.

5 1 or 2

6 3 or 4

7 5 and 6
